# Prevalence and impact of remote and hybrid work in academic health sciences libraries

**DOI:** 10.5195/jmla.2024.1905

**Published:** 2024-10-01

**Authors:** David Petersen, Matthew Covey, Janet Crum

**Affiliations:** 1 dpetersen@utmck.edu, Associate Professor, Senior Research & Learning Services Librarian and Assessment Coordinator, University of Tennessee Graduate School of Medicine; 2 mcovey@rockefeller.edu, University Librarian, Rita & Frits Markus Library, The Rockefeller University; 3 janetcrum@csufresno.edu, Dean of Library Services, Fresno State University, Fresno, CA

**Keywords:** Remote work, hybrid work, recruitment, retention, job satisfaction

## Abstract

**Objective:**

This study assesses the prevalence, usage, and impact of remote/hybrid work in academic health science libraries in 2022 and 2023. Due to differences in survey distribution, we focus primarily on the results of the second survey.

**Methods:**

Researchers surveyed administrators at Association of Academic Health Sciences Libraries (AAHSL) member libraries in the United States in March 2022 and library staff at academic health sciences libraries in March 2023.

**Results:**

The first survey received 71 responses that met inclusion criteria. Ninety-five percent of respondents indicated that remote/hybrid work was allowed in their libraries. Majorities indicated that remote/hybrid work had a positive impact on morale (86%), recruitment (53%) and retention (67%). The second survey received 383 responses that met inclusion criteria. 78% of respondents indicated they were allowed to work remotely, and majorities indicated remote/hybrid work positively impacted work/life balance (75%), morale/job satisfaction (69%), likelihood of staying at their current institution (64%), and productivity/overall effectiveness (58%). Respondents were less likely to accept a fully onsite (45% unlikely) or fully remote (20% unlikely) position than a hybrid one (1% unlikely). In a list of 9 factors associated with recruitment, retention, and job satisfaction, only salary and benefits ranked higher than remote/hybrid work.

**Conclusions:**

Remote/hybrid work is common in academic health science libraries and highly valued by employees. While not without challenges, remote/hybrid work appears to be a valuable tool to support recruitment, retention, and job satisfaction of workers in academic health sciences libraries. The findings of this study can inform library decision makers about future use of remote/hybrid work.

## INTRODUCTION

The COVID-19 pandemic caused major work disruptions and changes in the United States, including the large migration to remote and/or hybrid work during the pandemic. As the pandemic fades, remote/hybrid work is still available to many white-collar employees. In the broad job market, McKinsey's American Opportunity Survey (2022) noted that 58% of Americans can work remotely at least one day per week, and 87% of respondents indicated they would take advantage of that option if permitted [1]. Gallup reported that as of September 2023 the average worker in the United States works remotely 3.8 days per month [2].

Many libraries, including academic health sciences libraries, are currently trying to determine if and how to offer remote or hybrid work to their employees on a long-term basis. To inform those decisions, we conducted a survey in 2022 of academic health sciences library administrators and a 2023 survey of academic health sciences library staff. These surveys focus on the state of remote and hybrid work in academic health sciences libraries as well as its impact on factors that may influence recruitment and retention of employees, including those from historically underrepresented groups. The survey results may inform best practices for advocating for, implementing, and supporting remote/hybrid work in academic health sciences libraries.

Library literature related to remote work can be divided into two general time periods: before and after the onset of the COVID-19 pandemic in early 2020. While somewhat uncommon in libraries before the COVID-19 pandemic [3–5], remote work and other forms of flexible scheduling were discussed in the library literature as early as 1984. In fact, pre-COVID literature on remote work identifies many of the advantages and challenges discussed today such as increased job satisfaction, accommodations for employees with disabilities, home workspaces, and potential isolation [6–8]. As technology advanced, libraries began experimenting with telecommuting and even the National Library of Medicine provided a hybrid work program [9]. In 1992, the Association of Research Libraries (ARL) published the results of a survey of ARL member libraries on flexible work arrangements. Of the 89 respondents, 81 reported offering some type of flexible work arrangement but only 14 offered telecommuting/home-based work. The report's authors noted that “[l]ibrary managers have the unique opportunity to gain a competitive advantage by addressing the developing needs of the work force of the 21st century” [5]. We see similar arguments today in favor of flexible and remote/hybrid work.

In the first decade after the new millennium, several articles discussed communication tools and new developments in videoconferencing, online chat, and social networking facilitated by increased implementation of broadband internet that would transform remote work [10–12]. An accelerating shift to electronic collections also made remote work more feasible for librarians [13]. The increased interest in remote work was also reflected by two articles addressing ethnographic research on information maintained by four fully remote library workers and a proposed theoretical framework for remote's work future among librarians [14, 15].

Many articles were published in the library literature during the COVID-19 pandemic, particularly in 2020 and 2021, that described how individual libraries or departments within libraries coped with the shift to remote work brought on by the COVID-19 pandemic; however, we focused on articles reporting research results. Few published studies focus on academic health sciences libraries. Ragon, et al. conducted three surveys of academic health sciences library leaders to capture the status of the libraries and their services “at key points of the pandemic” [16]. Miller and Janke interviewed academic nursing librarians in Canada regarding how the pandemic affected their work, including (though not emphasizing) the impact of remote work [17]. Petersen investigated “two questions: whether remote and/or hybrid work arrangements were advertised in health sciences libraries prior to the COVID-19 pandemic, and whether there had been an increase in job postings that included flexible work arrangements during 2021–2022” [18]. Several studies have been conducted in general academic and public libraries regarding the prevalence, benefits, and drawbacks of remote work with results indicating productivity in the remote environment and benefits for recruitment and retention [3, 19–21].

## METHODOLOGY

Numerous terms have been used to describe remote/hybrid work. In this paper, we use the following terms: fully remote work (no in-office requirements), hybrid work (at least one day per week can be outside the office), and fully onsite (remote work not permitted or permitted in rare/special circumstances only). Finally, we use the term “flexible work arrangements” to refer to the broader concept of deviation from traditional five-day, forty-hour onsite work schedules. Employers who offer other flexible work arrangements (e.g., flex time, compressed work weeks, job sharing) may-but do not always-permit remote/hybrid work.

We designed two surveys to investigate the use of remote and hybrid work in academic health science libraries. The surveys featured questions to obtain data on the usage and prevalence of remote/hybrid work along with demographic questions that allowed us to explore respondents' answers by geographic region, longevity at institution, and by position category. We used Qualtrics to survey administrators of academic health sciences libraries in the United States in March 2022 and academic health science library employees at all levels in March 2023. Both surveys were approved by each of the authors' respective Institutional Review Boards (University of Arizona Institutional Review Board, # STUDY00000747, 2022–2023. The Rockefeller University Institutional Review Board, # 363385; University of Tennessee Graduate School of Medicine in Knoxville. RB number # 4893, 2022–2023). The 2022 survey contained eighteen multiple choice, ranking, and free response questions that asked about the status of remote and hybrid work before and during the pandemic and whether respondents believed remote/hybrid work would continue beyond the pandemic. This survey was distributed to the AAHSL-all email list, which includes directors and other senior administrators at AAHSL member libraries and remained open for three weeks. Respondents had to be a current director or senior administrator at an AAHSL library in the United States to be included in the data.

In 2023, we wanted to learn the extent to which remote/hybrid work persisted in academic health sciences libraries and to expand the survey population beyond administrators to include full-time employees of academic health sciences libraries in the United States. We designed a 25-question survey in Qualtrics using a mix of multiple choice, ranking, and free response questions and distributed it to several email lists including AAHSL-all, MEDLIB-L, MLA caucus and chapter email lists. It remained open for three weeks. The inclusion criteria were that the respondent was a current employee at an academic health sciences library.

At the conclusion of the surveys, results were stored and analyzed in Qualtrics. We ended the survey by asking respondents, “Is there anything else you would like to tell us about remote work policies and practices in your library?” The free text responses were coded by one researcher and verified by another. Responses were coded into the following categories: availability of remote work, effect on moral/productivity, effect on library operations, and effect on patron engagement. Once all responses were categorized, we analyzed them to identify themes and found the following themes were commonly expressed: positive and negative comments about remote/hybrid options, criticism about the implementation and execution of remote/hybrid work at their institutions, recruitment and retention, and equity concerns.

## RESULTS

In the 2022 AAHSL administrators survey, we received 81 total responses, of which 71 met inclusion criteria. Of respondents selecting a region (n = 65), a plurality of responses was received from the Northeast (n = 21, 32%) followed by the Midwest (n = 15, 23%), Southeast (n = 14, 22%), West (n = 11, 17%), and Southwest (n = 6, 9%). This approximately mirrors the geographic distribution of AAHSL institutions (n = 163), of which 27% are in the Northeast, followed by the Midwest (23%), Southeast (23%), West (16%), and Southwest (12%). Most (n = 46/67, 69%) had worked at their current institution for at least 8 years. Respondents were asked about current remote/hybrid work policies at their library as well as the expected continuation of such policies. The majority of respondents (n = 59/62, 95%) indicated the existence of hybrid work of at least 1–2 days offsite while 29% (n = 18/62) indicated that fully remote work was an option. Respondents were optimistic that remote/hybrid work would continue, with 55% (n = 35/64) viewing its continuation as extremely likely and 28% (n =18/64) as somewhat likely. The survey asked respondents to indicate the impact, either positive, neutral, or negative, of their library's remote/hybrid work policy on several items. Responses indicated that remote/hybrid work at their institution had a positive impact (86%, n = 55/64) on employee morale, retaining existing librarians (68%, n = 43/63) and recruiting new librarians (53%, n = 33/62); however, the majority of respondents had a neutral view of remote/hybrid work's impact on employee scholarship (57%, n = 36/63).

**Figure 1:**
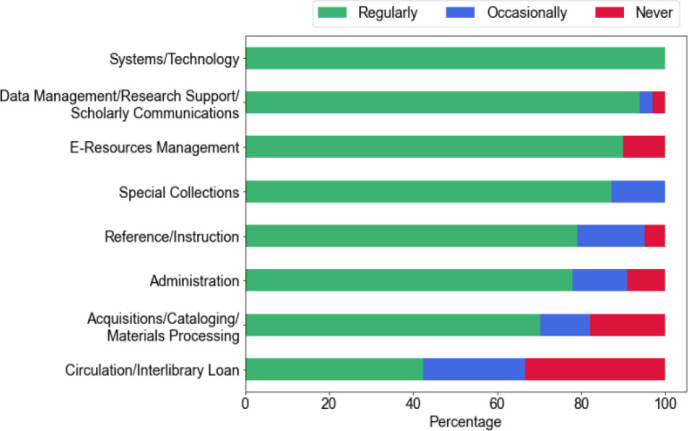
Current availability of remote/hybrid work by work area

For the 2023 survey of all academic health sciences library staff, we received 410 responses, of which 383 met inclusion criteria. When asked what best represented their role, 37% (n = 132/357) selected administrative options (directors, deputy directors, unit heads), 50% (n = 179/357) selected librarian, and 11% (n = 41/357) selected library staff. Forty-six percent of respondents (n = 164/357) indicated that they supervised someone. A plurality (n = 156/358, 44%) indicated that reference/instruction best represented their work area; a full breakdown of respondents by work area is shown in Figure 1. A plurality of responses (n = 111/358, 31%) came from the Northeast, followed by the Southeast (n = 97/358, 27%), Midwest (n = 66/358, 18%), West (n = 56/358, 16%) and Southwest (n = 28/358, 8%). Both public (n = 194/357, 54%) and private (n = 163/357, 46%) institutions were represented. Respondents also had a wide range of longevity at their current institution; one-third (n = 120/358) had spent fewer than 3 years while 31% (n = 111/358) had stayed longer than 12 years.

When we asked respondents to indicate their race, we received 346 responses, of which 284 (82%) identified as White, 14 (4%) identified as Black or African-American, 12 (3%) identified as Hispanic or Latino/a/x, 5 (1%) identified as Multiracial, 2 (0.6%) identified as Native American or Alaska Native, and 19 (5%) declined to answer. Because of the small number of respondents identifying as other than White (33, 10%), it was not possible to perform a meaningful analysis of survey results by race.

Respondents were asked several questions pertaining to remote/hybrid work. As expected, the frequency of remote work appeared to increase dramatically with the onset of the COVID-19 pandemic. Thirteen percent (n = 46/353) indicated they were allowed to work remotely on a regular basis prior to the pandemic, while 23% (n = 81/353) did so occasionally. Nearly all respondents indicated they worked remotely during the pandemic. Circulation/interlibrary loan respondents were the only group in which greater than one percent of respondents indicated they could not work remotely during the pandemic.

When asked if they were currently allowed to work remotely (fully or hybrid), 78% (n = 275/353) indicated that they did so on a regular basis, while 13% (n = 47/353) said they did but only for exceptional circumstances. Nine percent (n = 31/353) reported they did not have a remote/hybrid option. Eighty-one percent (n = 285/350) believed remote/hybrid work was somewhat likely or extremely likely to continue at their institution beyond the COVID-19 pandemic era.

**Figure 2:**
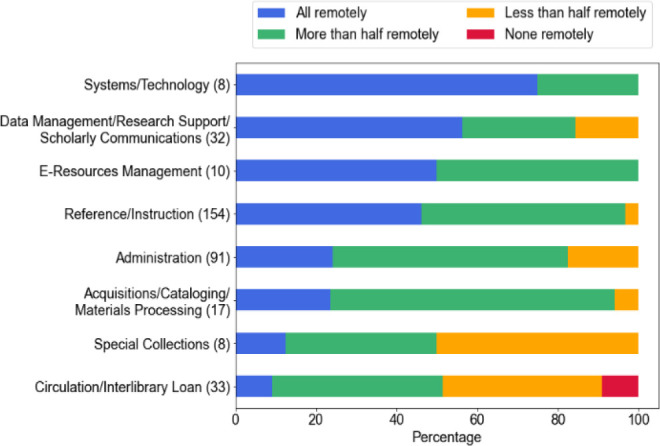
Library staff by work area: how much of your duties could you perform remotely?

We asked respondents to indicate to what extent they could perform their duties remotely (if allowed). Thirtyseven percent (n = 130/353) indicated they could perform all duties remotely, while 50% (n = 176/353) indicated that they could perform more than half of their duties remotely. Twelve percent (n = 44/353) said they could perform less than 50% of their position duties remotely while 0.8% (n = 3/353) said they could not perform any of their duties offsite. These results were further broken down by library work area (see Figure 2). Special collections (50%, n = 4/8) and circulation/interlibrary loan (39%, n = 13/33) had a higher percentage that believed less than half their work could be completed remotely.

We also wanted to examine the impact of remote/hybrid work policies on several important workplace issues. The majority of respondents (n = 260/345, 75%) said that their library's remote/hybrid work policy positively impacted their ability to balance work with family or other non-work responsibilities. Majorities of respondents also indicated that these policies had a positive impact on morale and job satisfaction (n = 239/345, 69%), likelihood of staying at current institution (n = 220/345, 64%), and work productivity and overall effectiveness (n = 201/345, 58%). Responses were mixed about the impact of remote/hybrid work on a respondent's scholarship; 38% (n =132/344) indicated a positive impact, while 56% (n = 193/344) were neutral. This positive impact from remote/hybrid work opportunities, however, did not extend to relationships and ability to collaborate with colleagues; 30% (n = 104/345) reported a positive impact, while 49% (n = 170/345) expressed a neutral opinion and 21% (n = 71/345) reported a negative impact. Respondents who had been employed at their institutions longer than 12 years more frequently indicated a negative impact on their relationships and collaboration with colleagues (n = 37/106, 35%) than those employed at their institutions for 0–3 years (n =14/117, 12%). Additional study is needed to determine how remote/hybrid work policies impact workplace relationships and collaboration in academic health sciences libraries.

**Figure 3:**
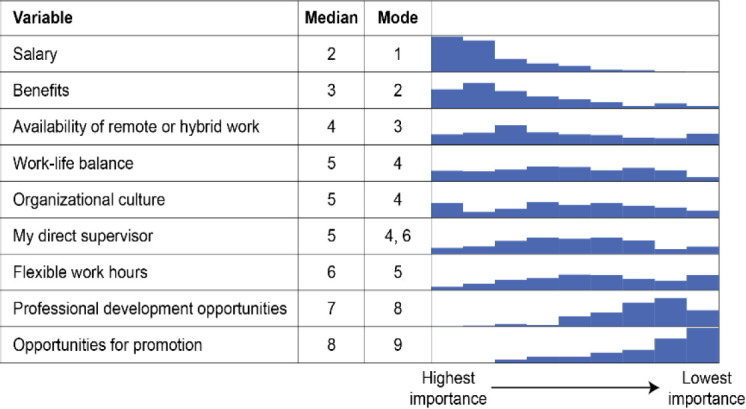
Job factors ranked by importance

We asked respondents to rank nine factors frequently considered to be beneficial to recruitment, retention, and satisfaction, as shown in Figure 3. The data suggest that respondents ranked remote and/or hybrid work only behind salary and benefits.

Given this high ranking, it was not surprising to find that respondents valued this option when assessing employment opportunities. 17% (n = 57/336) of respondents indicated they were very likely to accept a fully onsite position, while 45% (n = 151/336) indicated that they were not likely to do so. While almost all respondents (n = 339/344, 99%) were likely or somewhat likely to take a hybrid position, 20% (n = 66/338) were not likely to accept a position that was fully remote. These results varied somewhat by region. 12% (n = 13/107) of respondents in the Northeast indicated they would be very likely to take a fully in-person position compared to 26% (n = 7/27) in the Southwest. A majority of respondents in the West (n = 31/54, 57%), Southeast (n = 52/94, 55%), and Northeast (n = 54/107, 50%) indicated that they were very likely to take a fully remote position as compared to the Southwest (n =11/27, 41%) and Midwest (n = 29/64, 45%).

**Figure 4:**
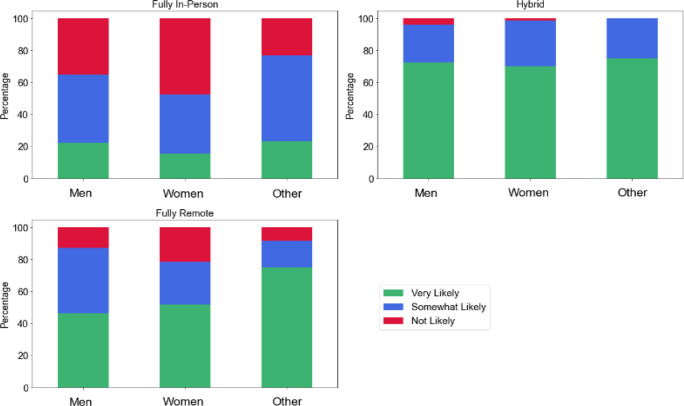
How likely are you to take a position with the following options? “Other” includes the following options from the survey: agender, genderqueer or genderfluid, nonbinary, questioning, other gender identity not listed. Fully In-Person: Men (n = 54), Women (n = 260), Other (n = 13); Hybrid: Men (n = 54), Women (n = 268), Other (n = 12); Fully Remote: Men (n = 54), Women (n = 263), Other (n = 12)

Responses to this question also varied by gender, as shown in Figure 4. Of the 346 respondents who selected a gender option, 78% (n = 269) were female, 16% (n = 54) were male, 4% (n = 13) selected another gender identity, and 3% (n=10) selected “prefer not answer.” We offered several options for indicating gender identity, but due to the small number of respondents who chose an option other than “male” or “female,” we collapsed those options into a single category. This was done, in part, to protect respondents' privacy. For respondents identifying as female, 46% (n =124/269) said they were not likely to take a fully on-site position, compared to 35% (n = 19/54) of male respondents and 23% (n = 3/13) of those who indicated another gender identity. Over three-fourths of respondents who indicated a gender identity other than “male” or “female” indicated they were very likely to take a fully remote position (n = 9/13, 69%), higher than female (n = 136/269, 51%) or male (n = 25/54, 46%) respondents. One should exercise caution when interpreting the results given the uneven groups and potential for confounders.

We also investigated the usage and popularity of remote/hybrid work options among people with disabilities. In response to a survey question, approximately 17% of respondents (n = 58/345) indicated that they identified as a person with a disability or other chronic condition. When asked to rank nine factors frequently considered to be beneficial to recruitment, retention, and satisfaction, respondents with disabilities identified salary, benefits, and remote/hybrid work options as their top three items, the same as all respondents. While we were not able to establish statistical significance, the results suggest that respondents with disabilities may be more inclined to accept a fully remote position (66%, n = 38/58) compared to all respondents (48%, n = 131/271) and may be less inclined to accept a fully onsite position (55% (n = 32/58) vs. 42% (n = 114/271) of those not identifying as having a disability). The low number of respondents identifying as a person with disabilities indicates that caution should be exercised when interpreting these results.

We ended the survey by asking respondents, “Is there anything else you would like to tell us about remote work policies and practices in your library?” We received 141 responses to this question. Several comments mentioned themes that were noted in articles referenced in the literature review. While the pandemic created an unprecedented opportunity for remote/hybrid work, many of the concerns, criticisms, and positive evaluations closely resemble those from articles written prior to the pandemic.

Unsurprisingly, there were many positive comments about remote/hybrid work. Several comments mention increased productivity and flexibility along with a few noting that they wish these options had been available when they had children or caregiving responsibilities. Others commented that remote/hybrid options had been “life-altering” with one commenter indicating they “would be forced to retire” if a remote option did not exist. Interestingly, some respondents indicated that remote/hybrid work enables them to better serve their patrons; one commented that “…few people want to meet in person. Faculty and students want to meet virtually.” Another positive facet of remote/hybrid work for some respondents was their mental health. One comment mentioned, “[remote/hybrid work] had a positive impact on both my work and interpersonal relationships as well as my mental health.” Another said, “[s]ince working from home during COVID, my anxiety has almost resolved itself.”

Several comments focused on recruitment and retention. Respondents said, “I will not take a job that requires fully in-person/onsite work again” and “[t]he reason that I left my previous job is because they wanted us to be in person full time…I refuse to go back to full time in person work.” Others noted that “[t]he flexibility that hybrid work provides is the only thing keeping some of our library personnel from seeking opportunities elsewhere,” “[p]eople in my library see remote work as a benefit now,” and “several job candidates declined job offer[s] due to no remote/hybrid policy.” One respondent took a more nuanced approach: “[t]here is suspicion that remote work policies are being used as a substitute for other career needs of employees, almost as if a hybrid schedule is supposed to make up for lack of organizational commitment in other areas.”

Respondents were not uniformly positive about remote/hybrid work. Negative comments indicated that some people “prefer to work on campus for interaction with colleagues,” “feel isolated and disconnected” while working remotely and view remote/hybrid work as “crippl[ing] employee relationships.” Others wanted to keep “work and home life separate” and noted that they “work more reasonable hours when not working from home.” Another respondent commented that with a hybrid work environment, the office “feels empty” and they “miss in-person interactions.” One comment mentioned that hybrid work has not generally helped that institution's staff and observes “extremely high levels of burnout, not enough personnel, and a lack of promotion or financial incentives for the work being done.”

Perhaps the largest topic covered in the comments concerned equity issues. While many respondents indicated general approval of remote/hybrid work, several had serious concerns about how it was being implemented at their institutions. Respondents noted that “remote work is not applied equitably” and that “remote work provides the greatest monetary advantage to professional staff who are highest earners.” Another said, “[a]llowing some staff to work remotely is negatively affecting the morale of those of us who have to work on-site… It's unfair and creates a divide between departments.” Recognizing the division between library faculty/professional staff and library staff, one respondent noted, “I don't like the feeling of requiring the lowest paid employees to be here more than other employees just due to the nature of their job.” Going further, another commenter said, “[m]y university's policy has been inequitable and inconsistent. We are no longer allowed remote work except until [sic] special circumstances that must be documented. At the same time, we no longer have weather closures and are told to work from home on the days that the campus is closed. This is terrible for employee morale and frankly is responsible for a definite increase in quiet quitting in our organization.”

Several comments focused on positive actions to correct inequities related to remote/hybrid work. Figure 1 illustrates that circulation/interlibrary loan staff were the least likely to have remote/hybrid work options. One respondent noted, “Our Access Services team is not working remotely anymore; but we were able to offer them a shorter work week (35 hours over 4 days [versus] 35 hours over 5 days) when we phased out remote work for them.” Another commenter in Access Services said that a remote/hybrid work “option reduces tensions between faculty and staff and makes me feel valued (not just a body at a desk).” These comments indicate that employers can help workers feel valued by offering flexibility and ensuring that all employees benefit from remote/hybrid work.

### Discussion

The COVID-19 pandemic greatly increased the possibility of remote/hybrid work for many employees in academic health sciences libraries as survey results indicated that more than three-fourths of respondents are able to regularly work a remote/hybrid schedule.

It is also worth noting that though remote/hybrid work was often treated as a novelty during the pandemic, many of the benefits and drawbacks were identified in the library literature decades ago, as indicated in the introduction [5–6]. Some respondents indicated similar feelings to the pre-pandemic library literature on remote/hybrid work noting that the disconnect and isolation could be difficult while also appreciating the flexibility that the arrangements provided. The COVID-19 pandemic merely forced most libraries into a remote and/or hybrid work environment in an abrupt manner. One challenge will be the revision, implementation, and sustainability of remote and/or hybrid work policies. Despite this, libraries have an opportunity to integrate remote and/or hybrid work to benefit staff and the library through flexible work schedules and newly imagined position responsibilities.

Our results suggest that health sciences library workers value remote/hybrid work highly, and that respondents feel the availability of remote/hybrid work positively affects recruitment, retention, work-life balance, morale/job satisfaction, and productivity. However, respondents' assessments were mixed about the impact of remote/hybrid work on other areas, including relationships and collaboration with colleagues. Longer serving colleagues expressed dissatisfaction three times more frequently with remote/hybrid work than their more newly hired colleagues. It remains to be seen how remote/hybrid options will impact collaboration and relationships over the longer term. This result invites further research into how library staff form and maintain relationships, collaborate effectively, and maintain a cohesive team atmosphere in a remote/hybrid environment.

The data suggest some interesting possibilities that invite reflection and further research. First, substantial percentages of respondents were not inclined to take either a fully in-person position or a fully remote position. These responses varied by gender. Female respondents more frequently indicated that they were unlikely to accept a fully in-person work option compared to male respondents; however, respondents with a gender identity other than male or female more frequently indicated a desire for a fully remote position. Results were mixed as to whether underrepresented groups prioritized remote/hybrid work to a greater degree than the overall sample. This data suggests that health sciences library workers desire flexibility, but many are unwilling to completely isolate themselves from in-person work. Of course, during the height of the pandemic, many worked in a fully remote capacity. However, as the pandemic recedes, and a new normal develops, people may be uncertain what constitutes their ideal work environment.

Second, recruitment and retention will remain major factors influencing-and being influenced by-the availability of remote/hybrid work. The survey data strongly suggests that a hybrid schedule is valued and very few respondents indicated a negative response to such an offering. Indeed, a 2023 survey of US workers found that 67% would be willing to take a pay cut if they could retain a hybrid schedule [22]. However, several comments suggested that an inequitable implementation of remote/hybrid work can lead to resentment, division, and disengagement. Our results suggest that institutions offering remote/hybrid work may attract additional applicants when conducting job searches, while institutions requiring fully onsite work may have difficulty recruiting and retaining employees.

This study has several limitations. The sample was self-selected and represents only two moments during the aftermath of the pandemic lockdown during 2020–2021. Researchers did not have data on how many health sciences library staff work in the United States. Thus, they were unable to very if respondents adequately represented all regions within the United States. The survey's respondents were 78% female, 16% male, and 4% nonbinary; this roughly approximated Pionke's 2020 study of the Medical Library Association that found 79% of respondents identified as female while 13% identified as male [23]. A 2023 Bureau of Labor Statistics report found that 82.5% of librarians and media collections specialists identified as female [24]. Additionally, our survey data varied from a recent survey of the Medical Library Association regarding race or ethnicity with our survey having higher participation from White respondents.

Remote/hybrid work is a complex issue; its impact and success can be highly dependent on institutional setting, mix of employees, and/or specifics of implementation. Indeed, the term “hybrid work” can be ambiguous. People working remotely four days per week may have different opinions than those that work remotely one day per week. Unfortunately, the data did not allow researchers to make statistically supported assertions about the preference and experience of library staff from marginalized backgrounds. While data indicated that most academic health sciences libraries offer some form of hybrid work, we did not determine whether this still held true in a hospital or special library environment. Many facets of remote/hybrid work in health science libraries need further exploration including examining librarians from marginalized backgrounds and their experiences with remote/hybrid work, preference for remote/hybrid work based on the age of library staff, and the impact of remote/hybrid work on feelings of community in the workplace.

## CONCLUSION

Data from this study indicate that the pandemic initiated a large shift in academic health sciences libraries offering remote/hybrid work arrangements and that work-from-home options show a positive impact on a person's desire to apply for or stay at a position. Remote/hybrid work options are important to employees, outranked only by salary and benefits. While many respondents value a remote/hybrid work option, it must be clearly and equitably implemented to avoid resentment, low morale, and disengagement.

Advocating for remote/hybrid work options is critical and involves many groups including library leadership, librarians, library staff, unions, and professional associations. Survey responses indicate that careful emphasis should be placed on the equitable and transparent implementation of remote/hybrid work. In addition to facilitating in-person work, library leaders will need to be intentional about creating a virtual environment that fosters collaboration, innovation, and trust. Survey results suggest that remote/hybrid work may continue to be a feature of the health sciences library workplace for the foreseeable future.

## Data Availability

Data associated with this article are available in the Open Science Framework at: https://osf.io/d6g2r/.

## SUPPLEMENTAL FILES

**Appendix A:** 2022 Survey Instrument

**Appendix B:** 2023 Survey Instrument
