# Supplementary material for: Prevalence and impact of remote and hybrid work in academic health sciences libraries
Source: J Med Libr Assoc. 2024 Oct 7;112(4):298–306. doi: 10.5195/jmla.2024.1905 (PMC11486086; doi:10.5195/jmla.2024.1905)
Supplement: Supplementary file 1 — Appendix A: 2022 Survey Instrument [file jmla-112-4-298-s01.pdf]

IRB NUMBER: 4893
IRB APPROVAL DATE: 01/14/2022

Default Question Block

Analyzing Changes in Work-From-Home Policies for Academic Medical and Health Science Librarians

You are invited to complete this survey as part of a research project being conducted by David Petersen, MSLIS, MA, AHIP (University of Tennessee Graduate School of Medicine), Matthew Covey, PhD (The Rockefeller University), and Janet Crum, MLS, AHIP (University of Arizona). We are investigating remote/work-from-home policies at Association of Academic Health Sciences Libraries (AAHSL) institutions in the United States to identify trends and the impacts of those trends on library staff.

The survey is 14 questions long and will take approximately 7 minutes to complete. Completing the survey is voluntary. There are no known risks or personal benefits to completing this survey. You will be asked about your library's remote work policies before and during the COVID-19 pandemic, and you will have an opportunity to upload policy documents if you are comfortable doing so. The survey does not request personally-identifiable data, but any policy documents you upload could identify your institution unless you redact that information, and the combination of your institution and your other responses could enable someone to personally identify you. We will not share any personally-identifiable information with anyone else.

Please limit to one response per institution.

If you have any questions, please contact David Petersen, Assistant Professor, Research & Learning Services Librarian at: dpetersen@utmck.edu.

- ○ I consent to participate
- ○ I do not consent to participate

Are you a director or senior administrator in an academic health sciences library located in the United States?

- ○ Yes
- ○ No

Which of the following options best represents your title?

- ○ Dean or Director
- ○

IRB NUMBER: 4893
IRB APPROVAL DATE: 01/14/2022

Deputy or Associate Director

- ○ Other (please specify)

In which region of the United States is your library located?

- ○ Midwest - IA, IL, IN, KS, MI, MN, MO, ND, NE, OH, SD, WI
- ○ Northeast - CT, DC, DE, MA, MD, ME, NH, NJ, NY, PA, RI, VT
- ○ Southeast - AL, AR, FL, GA, KY, LA, MS, NC, SC, TN, VA, WV
- ○ Southwest - AZ, NM, OK, TX
- ○ West - AK, CA, CO, HI, ID, MT, NV, OR, UT, WA, WY

Is your institution

- ○ Public
- ○ Private

How long have you worked at your current institution?

- ○ 0-3 years
- ○ 4-7 years
- ○ 8-11 years
- ○ 12 years or longer

Did your institution permit fully remote work or hybrid (remote + in-person) schedules for library employees *prior to* the COVID-19 pandemic?

- ○ Yes for most/all library employees (including part-time, temporary, and/or student workers)
- ○ Yes for most/all library employees (excluding part-time, temporary, and/or student workers)
- ○ Yes for employees in selected job categories or positions
- ○ Yes for temporary/special cases only
- ○ No

Did your institution permit fully remote work or hybrid (remote + in-person) schedules for library employees *at any point during* the COVID-19 pandemic?

- ○ Yes for most/all library employees (including part-time, temporary, and/or student workers)
- ○ Yes for most/all library employees (excluding part-time, temporary, and/or student workers)
- ○ Yes for employees in selected job categories or positions
- ○ Yes for temporary/special cases only

IRB NUMBER: 4893
IRB APPROVAL DATE: 01/14/2022

○ No

Does your institution *currently* permit fully remote work or hybrid (remote + in-person) schedules for library employees?

○ Yes for most/all library employees (including part-time, temporary, and/or student workers)

○ Yes for most/all library employees (excluding part-time, temporary, and/or student workers)

○ Yes for employees in selected job categories or positions

○ Yes for temporary/special cases only

○ No

Which of the following options are available to employees in your library?

☐ Fully remote work

☐ Hybrid schedule (remote 1 or 2 days per week)

☐ Hybrid schedule (remote 3 or 4 days per week)

How likely is your institution/library to continue allowing remote or hybrid work arrangements after the COVID-19 pandemic?

	Extremely unlikely	Somewhat unlikely	Neither likely nor unlikely	Somewhat likely	Extremely likely
Likelihood of allowing remote or hybrid work after the COVID-19 pandemic	○	○	○	○	○

To which entity does the health sciences library report?

○ University administration (e.g., provost)

○ University library/library system

○ College/School of Medicine

○ Other (please specify)

Which parties were involved in developing your library's remote/hybrid work policy? (check all that apply)

☐ University administration

☐ University human resources

☐ College of Medicine administration and/or human resources

☐ Library administration (e.g., director, deputy director)

☐ Library human resources

IRB NUMBER: 4893
IRB APPROVAL DATE: 01/14/2022

- ☐ Library leadership team
- ☐ Librarians/library faculty
- ☐ Library staff (other than librarians/faculty)
- ☐ Other (please specify)

Please indicate the impact of your library's remote/hybrid work policy on each item listed below.

	Negative impact	Neutral/no impact	Positive impact
Employee morale	○	○	○
Recruiting new librarians	○	○	○
Recruiting new staff (other than librarians)	○	○	○
Retaining existing librarians	○	○	○
Retaining existing staff (other than librarians)	○	○	○
Employee scholarship (research, publications, presentations)	○	○	○

Is there anything else you would like to tell us about remote work policies and practices in your library?

As part of this study, we would like to analyze remote work policies from libraries to identify common elements and trends. Would you please share your library's and/or university's policies with us?

If your library's and/or university's policies are publicly available on the web, please share the link(s) here.

If your library's and/or university's policies are not on the web, please upload them below.

Upload policy document(s)

Thank you very much for completing this survey! Your responses will help us better understand remote work practices in US academic health sciences libraries and provide valuable information for health sciences library leaders and staff.

IRB NUMBER: 4893
IRB APPROVAL DATE: 01/14/2022

Survey Powered By Qualtrics
