# Supplementary material for: Prevalence and impact of remote and hybrid work in academic health sciences libraries
Source: J Med Libr Assoc. 2024 Oct 7;112(4):298–306. doi: 10.5195/jmla.2024.1905 (PMC11486086; doi:10.5195/jmla.2024.1905)
Supplement: Supplementary file 2 — Appendix B: 2023 Survey Instrument [file jmla-112-4-298-s02.pdf]

Default Question Block

Analyzing Changes in Work-From-Home Policies for Academic Medical and Health Science Librarians

You are invited to complete this survey as part of a research project being conducted by David Petersen, MSLIS, MA, AHIP (University of Tennessee Graduate School of Medicine), Matthew Covey, PhD (The Rockefeller University), and Janet Crum, MLS, AHIP (University of Arizona). We are investigating remote/work-from-home policies at academic health sciences libraries in the United States to identify trends and the impacts of those trends on library staff.

The survey is 25 questions long and will take approximately 10 minutes to complete. Completing the survey is voluntary. There are no known risks or personal benefits to completing this survey. You will be asked about your current role, your library's remote work policies, and the impact of those policies and other factors on your work life. The survey does not request personally-identifiable data, but the combination of your institution and your other responses could enable someone to personally identify you. We will not share any personally-identifiable information with anyone else.

If you have any questions, please contact David Petersen, Assistant Professor, Research & Learning Services Librarian, at: dpetersen@utmck.edu.

For questions about your rights as a participant in this study or to discuss other study-related concerns or complaints with someone who is not part of the research team, you may contact the University of Arizona Human Subjects Protection Program Director at 520-626-8630 or online at https://research.arizona.edu/compliance/human-subjects-protection-program.

- ○ I consent to participate
- ○ I do not consent to participate

Are you an employee of an academic health sciences library located in the United States?

- ○ Yes
- ○ No

In which region of the United States is your library located?

- ○ Midwest - IA, IL, IN, KS, MI, MN, MO, ND, NE, OH, SD, WI
- ○ Northeast - CT, DC, DE, MA, MD, ME, NH, NJ, NY, PA, RI, VT
- ○ Southeast - AL, AR, FL, GA, KY, LA, MS, NC, SC, TN, VA, WV
- ○ Southwest - AZ, NM, OK, TX
- ○ West - AK, CA, CO, HI, ID, MT, NV, OR, UT, WA, WY

Is your institution

- ○ Public
- ○ Private

How long have you worked at your current institution?

- ○ 0-3 years
- ○ 4-7 years
- ○ 8-11 years
- ○ 12 years or longer

Which of the following options best represents your work area?

- ○ Administration
- ○ Reference/Instruction
- ○ Circulation/Interlibrary Loan
- ○ Acquisitions/Cataloging/Materials Processing
- ○ Systems/Technology

○ E-Resources Management

○ Data Management/Research Support/Scholarly Communications

○ Special Collections

What percentage of your time/FTE is typically allocated to each of the following areas? (Total must add up to 100%)

Administration	0	%
Reference/Instruction	0	%
Circulation/Interlibrary Loan	0	%
Acquisitions/Cataloging/Materials Processing	0	%
Systems/Technology	0	%
Electronic Resources Management	0	%
Data Management/Research Support/Scholarly Communications	0	%
Special Collections	0	%
**Total**	0	%

Which of the following options best represents your role?

○ Dean or Director

○ Deputy or Associate Director

○ Department or unit head

○ Librarian

○ Library staff

○ Other (please specify)

Does your position require an MLS or other graduate degree?

○ Yes

○ No

Do you directly supervise anyone?

- ○ Yes
- ○ No

What best represents your position line?

- ○ Tenure track
- ○ Multi-year appointment
- ○ Annual appointment
- ○ No contract

Is your position union represented--part of a collective bargaining agreement?

- ○ Yes
- ○ No

To what extent could you perform all the duties of your position remotely (if allowed to do so)?

- ○ I could perform all duties remotely
- ○ I could perform more than half of my duties remotely
- ○ I could perform less than half of my duties remotely
- ○ I couldn't perform any of my duties remotely

Was your position allowed to work remotely (fully remote and/or hybrid) prior to the COVID-19 pandemic?

- ○ Yes - on a regular basis
- ○ Yes - only occasionally
- ○ No
- ○ Not Sure

Was your position allowed to work remotely (fully remote and/or hybrid) at any time during the COVID-19 pandemic?

- ○ Yes - on a regular basis
- ○ Yes - only occasionally
- ○ No

Are you *currently* allowed to work remotely (fully remote work and/or hybrid)?

- ○ Yes - on a regular basis
- ○ Yes - only occasionally (e.g., due to weather or illness)
- ○ No

Which of the following options are available to you (check all that apply)?

- ☐ Fully remote work
- ☐ Hybrid schedule (remote 1 or 2 days per week)
- ☐ Hybrid schedule (remote 3 or 4 days per week)

How likely is your institution/library to continue allowing remote or hybrid work arrangements after the COVID-19 pandemic?

	Extremely unlikely	Somewhat unlikely	Neither likely nor unlikely	Somewhat likely	Extremely likely
Likelihood of allowing remote or hybrid work after the COVID-19 pandemic	○	○	○	○	○

Please indicate the impact of your library's remote/hybrid work policy on each item listed below.

	Negative impact	Neutral/no impact	Positive impact
My morale and job satisfaction	○	○	○
How likely I am to stay at my current	○	○	○

	Negative impact	Neutral/no impact	Positive impact
institution			
My relationships and ability to collaborate with my colleagues	○	○	○
My work productivity and overall effectiveness	○	○	○
My scholarship (research, publications, presentations)	○	○	○
My ability to balance work with family or other non-work responsibilities	○	○	○

If you were to look for a new position, how likely would you be to accept one if it was:

	Very Likely	Somewhat Likely	Not Likely
Fully in person/onsite	○	○	○
Hybrid	○	○	○
Fully remote	○	○	○

Please rank the following factors in order of importance to you, with 1 being the best. (*To rank the listed items, drag and drop each item.*)

Benefits

Salary

Availability of remote or hybrid work

Organizational culture

My direct supervisor

Professional development opportunities

Work-life balance

Opportunities for promotion

Flexible work hours

We are asking the following questions so we can compare anonymous responses across individuals and groups in order to understand the different experiences of people with different identities. We believe it is important to ask about these intersecting identities in case individuals from certain groups are having substantively different perceptions and experiences compared to individuals from other groups. Any identifying information shared via this survey will be removed before results are shared.

Which of the following best describes you?

- ○ Asian or Asian American
- ○ Black or African-American
- ○ Hispanic or Latino/a/x
- ○ Native American or Alaska Native
- ○ Native Hawaiian or Pacific Islander
- ○ White or Caucasian
- ○ Multiracial
- ○ Other race or ethnicity not listed here
- ○ Prefer not to answer

Gender

- ○ Agender
- ○ Female
- ○ Genderqueer or genderfluid
- ○ Male
- ○ Nonbinary
- ○ Questioning
- ○ Two-Spirit
- ○ Other gender identity not listed
- ○ Prefer not to answer

Do you identify as a person with a disability or other chronic condition?

- ○ Yes
- ○ No
- ○ Prefer not to answer

Do you currently have caregiving responsibilities for another person (e.g., a child, spouse, or parent)?

- ○ Yes
- ○ No
- ○ Prefer not to answer

Is there anything else you would like to tell us about remote work policies and practices in your library?

Thank you very much for completing this survey! Your responses will help us better understand remote work practices in US academic health sciences libraries and provide valuable information for health sciences library leaders and staff.

Survey Powered By Qualtrics
